# K205R specific nanobody-horseradish peroxidase fusions as reagents of competitive ELISA to detect African swine fever virus serum antibodies

**DOI:** 10.1186/s12917-022-03423-0

**Published:** 2022-08-20

**Authors:** Angke Zhang, Shuya Wu, Xiaohong Duan, Huijun Zhao, Haoxin Dong, Jiahui Ren, Mingfang Zhang, Jiaji Li, Hong Duan, Gaiping Zhang

**Affiliations:** 1 College of Veterinary Medicine, Henan Agricultural University, Zhengzhou, 450046 Henan China; 2 International Joint Research Center of National Animal Immunology, College of Veterinary Medicine, Henan Agricultural University, Zhengzhou, 450046 Henan China; 3 Hebei Station of Livestock Improvement, Shijiazhuang, 050061 Hebei China; 4 Key Laboratory of Animal Immunology of the Ministry of Agriculture, Henan Academy of Agricultural Sciences, Zhengzhou, 450002 Henan China

**Keywords:** Nanobody-HRP, ASFV, K205R, cELISA, Antibody

## Abstract

**Background:**

African swine fever virus (ASFV) is a highly contagious hemorrhagic disease and often lethal, which has significant economic consequences for the swine industry. Due to lacking of commercial vaccine, the prevention and control of ASF largely depend on early large-scale detection and screening. So far, the commercial ELISA kits have a long operation time and are expensive, making it difficult to achieve large-scale clinical applications. Nanobodies are single-domain antibodies produced by camelid animals, and have unique advantages such as smaller molecular weight, easy genetic engineering modification and low-costing of mass production, thus exhibiting good application prospects.

**Results:**

The present study developed a new method for detection of ASFV specific antibodies using nanobody-horseradish peroxidase (Nb-HRP) fusion proteins as probe. By using camel immunization, phage library construction and phage display technology, five nanobodies against K205R protein were screened. Then, Nb-HRP fusion proteins were produced using genetic modification technology. Based on the Nb-HRP fusion protein as specific antibodies against K205R protein, a new type of cELISA was established to detect ASFV antibodies in pig serum. The cut-off value of the cELISA was 34.8%, and its sensitivity, specificity, and reproducibility were good. Furthermore, the developed cELISA exhibited 99.3% agreement rate with the commercial available ELISA kit (kappa value = 0.98).

**Conclusions:**

The developed cELISA method has the advantages of simple operation, rapid and low-costing, and can be used for monitoring of ASFV infection in pigs, thus providing a new method for the prevention and control of ASF.

**Supplementary Information:**

The online version contains supplementary material available at 10.1186/s12917-022-03423-0.

## Background

African swine fever (ASF), which the caused pathogen is ASF virus (ASFV), is one of the most complex and economically devastating viral diseases in swine herds, classified as a notifiable disease by the World Organization for Animal Health (OIE) [1], and the morbidity and mortality rates is very high [2]. ASF. In August 2018, Shenyang City, Liaoning Province, China reported the first domestic case of ASF infection, and since then, it has spread across the country at an extremely fast rate, causing huge economic losses to the pig industry [3, 4]. There are no effective preventive vaccines and therapeutic drugs for this disease, and the effective measure to prevent the spread of the epidemic is early detection and culling [5]. Due to the lack of effective commercial ASF vaccine, the presence of antibodies in serum is a clear indicator of ASFV infection in pigs. Comprehensive and timely serological testing is critical for controlling viruses in infected herds and monitoring the absence of disease [6].

Enzyme-linked immunosorbent assay (ELISA), a traditional assays based on antigen–antibody specific immune responses, is a fast and convenient tool for detecting antigen or antibody levels in serum, and is considered the gold standard in the application in detection of viral infectious diseases [7]. Current OIE-approved assays for ASFV-specific antibodies detection include a large-scale initial screening of pig serum samples by OIE-ELISA, followed by western blot analysis to further confirm the results of suspicious and positive samples [8, 9]. In addition, Cao et al. established a competitive ELISA (cELISA) based on p54 protein as a coating antigen, which has comparable specificity, sensitivity, and coincidence rate with the commercial ELISA kit, however, this method is unable to distinguish low and high pathogenic ASFV strains [10]. Oviedo et al. establish an indirect ELISA using p30 as the coating antigen, which exhibiting the same sensitivity as the method recommended by OIE, but has higher specificity [11]. There are also some reports using p72, pp62 and other proteins to establish some ASFV serological diagnostic methods, indicating their potential as effective diagnostic tools for ASF [12, 13]. At present, the large-scale clinical ASFV serum antibody detection are mainly traditional indirect ELISA kit. These kits are widely used for detection of serum antibodies. However, higher purity antigens and enzyme-labeled secondary antibodies are required, and this also leads to higher production costs for commercial kits. Enzyme-linked antibodies are critical reagents for the development of sensitive and specific different ELISA methods, such as enzyme-linked monoclonal and polyclonal antibodies. However, traditional antibodies have some shortcomings limiting their application, such as monoclonal antibodies are costly and difficult to genetically manipulation, while polyclonal antibodies suffer from poor batch-to-batch stability. Hence, there is an urgent need to develop strategies to produce alternative scaffolds.

Nanobody (Nb) is a new type of genetically engineered antibody, also known as single-domain antibody, and it is obtained by cloning the variable region of heavy chain antibody naturally lacking light chain in camelid animals [14]. Compared with traditional antibodies, Nb has the advantages of small molecular weight, high affinity, high specificity and stability, good solubility, low immunogenicity, and strong penetrating ability, which is beneficial to be used as a new analytical reagent in immunochemistr [15–18]. As a new member of the antibody industry, Nb is small in size but has the complete antigen binding ability of the parent antibody, which is a beneficial complement to traditional antibodies.

ASFV is a large enveloped virus containing a double-stranded DNA (dsDNA) genome of 170–194 kbp [19, 20], it belongs to the ASFV family, the genus ASFV. Its genome contains approximately 160 major open reading frames (ORFs) and encoding products, including enzymes, structural proteins, and scaffolding proteins [21]. ASFV is currently the only DNA arbovirus, and no virus serologically related to ASFV has been identified to date. In infected macrophages, more than 100 virus-induced proteins have been identified, of which at least 50 can interact with infected pigs or recovered pigs of serum responses, 40 were able to bind to virions. K205R protein can induce a strong immune response in pigs, which is one of the main antigens of ASFV [22, 23]. As a protein expressed in the early stages of viral infection, K205R appears at as early as 4 h after infection, and localizes to the ASFV replication factories [2, 24]. Hence, the K205R protein possesses great implications for the early diagnosis of ASFV.

As a new type of antibody, nanobodies are simple to prepare and lower in cost, and are able to bind to specific epitopes that cannot be recognized by traditional antibodies [25]. In our previous studies, a fast and convenient platform has been constructed to produce different nanobody-horseradish peroxidase (Nb-HRP) fusion proteins, which was used as an ultrasensitive probe for detection PRRSV antibodies in pig serum [26]. In the present study, the specific nanobodies against K205R protein were isolated, and specific Nb-HRP fusion proteins were produced using the previously constructed platform. By using the Nb-HRP fusion protein as a sensitive probe, a cELISA for detection of ASFV antibodies in inactivated pig serum was established. A series of experimental results showed that the developed cELISA had high specificity and reproducibility, and it showed comparable agreement and sensitivity with the commercial ELISA kit, while the former takes less time, and thus has broad application prospects in clinical swine serum detection of ASFV antibodies.

## Materials and methods

### Cells, vectors, and sera

Human embryonic kidney cells (HEK-293 T) were cultured in Dulbecco's Modified Eagle's Medium (DMEM; Solarbio, China) supplemented with 10% fetal bovine serum (FBS, Gibco, Carlsbad, CA, USA) and 1% antibiotic–antimycotic (Life Technologies Corp) at 37 °C under 5% CO_2_. ASFV-K205R protein was expressed using the pET-30a prokaryotic expression vector (Novagen, USA). The VHH library was constructed using the pCANTAB 5E vector (GE healthcare Life Science, Pittsburgh, USA), and the Nb-HRP fusion protein platform was constructed using the pCAGGS-HA vector as the backbone plasmid.

One hundred forty-seven ASFV antibody-negative serums were collected from ASF decontamination pig farms, all serums were all confirmed ASFV antigen- and antibody-negative using RT-qPCR and commercial ELISA kit detection (Beijing JinnuoBaitai Biotechnology Co., Ltd.). At the same time, 285 clinical serum samples collected from different farms in Henan Province were detected using the developed cELISA and commercial ELISA kit. It is clearly stated that the pig serum used in this experiment is inactivated at 60 °C for 30 min.

### Expression and purification of recombinant K205R Protein

The K205R gene was amplified using PCR with primer pairs K205R-F and K205R-R (Table S1) using the synthesized K205R gene as the template (GENEWIZ Company, Jiangsu, China). Subsequently, the K205R gene was digested by enzymes *BamH* I and *Xho* I and then cloned into the pET-30a vector. The correctly sequenced plasmid was named pET-30a-K205R-His. Next, the pET-30a-K205R-His plasmids were transformed into *E.coli* BL21 (DE3) (TransGen Biotech, Beijing, China), a single clone was selected and treated with 0.5 mM isopropyl β-D-1-thiogalactopyranoside (IPTG) at 37˚C for 12 h. After sonication and centrifugation, K205R proteins in inclusion bodies were dissolved in 8 M urea and then purified using a Ni–NTA resin (Roche, Mannheim, Germany). The expression and purification of K205R protein was identified by SDS-PAGE, the antigenicity was analyzed by Western blot and iELISA.

### Bactrian camel immunization and library construction

Based on previously reported procedures, a adult male Bactrian camel was immunized five times subcutaneously using the purified K205R protein [27, 28]. Briefly described as following, 2 ml K205R protein (2 mg) was mixed with an equal volume of Freund's complete adjuvant (Sigma-Aldrich, Merck KGaA, USA) for the first immunization. For the subsequent immunizations, 2 ml K205R protein (2 mg) was mixed with equal volume of Freund's incomplete adjuvant (Sigma-Aldrich). The camel was immunized once every 2 weeks. To determine the success of immunization, after the fourth immunization, camel serum sample was isolated and iELISA was performed to determine the antibody titer against K205R protein.

One week after the fifth immunization, 200 ml of camel whole blood was collected and peripheral blood mononuclear cells (PBMCs) were isolated using Ficoll-Paque PLUS (Cytiva) with Leucosep™ tubes (Greiner Bio-One GmbH). Total RNA was extracted with 1 × 10^7^ PBMCs and then reverse transcribed into cDNA. Nested PCR was performed to amplify the VHH gene using the cDNA as template. The first round of PCR products (~ 700 bp) were amplified with the VHH-F1 and VHH-R1 primers (Table S1), using the first round PCR products as template, the second round of PCR (~ 400 bp) were amplified with primers VHH-F2 and VHH-R2. Finally, the VHH gene was inserted into the phage display vector pCANTAB 5E with *Not* I and *Pst* I enzyme sites (NEB, Ipswich, MA, USA), and transformed into fresh *E. coli* TG1 competent cells using an electroporation machine.

Cells were incubated overnight at 37 °C on LB agar plates containing 100 mg/ml ampicillin and 2% glucose. The colonies on the plates were scraped and resuspended in 3 ml of LB medium containing 20% (v/v) glycerol to prepare a VHH phage library against K205R protein, the library was stored at -80 °C.

### Screening and identification of ASFV K205R Nanobodies

As previously reported, phage display technology was used to screen anti-ASFV K205R protein specific Nbs by three rounds of bio-panning [26], and the M13K07 helper phage were utilized to rescue the VHH phage libraries. In bio-panning, 4 µg/well ASFV K205R protein was used as the coating antigen. The specific phage particles were significantly enriched after three rounds of bio-panning using anti-M13/HRP conjugate phage ELISA. Then, 96 single clones were randomly selected and induced to express soluble VHHs with E tag using 1 mM IPTG. Then, all the obtained soluble VHHs proteins were evaluated for their binding ability to ASFV K205R protein with iELISA using anti-E-Tag monoclonal antibody (GenScript, China). Finally, the positive clones identified by iELISA were sequenced and classified according to the sequence diversity in the third complementarity determining region (CDR3). Besides, the specificity and affinity of nanobodies screened were evaluated by iELISA using periplasmic extracts at different dilutions as primary antibodies.

### Expression of Nb-HRP against ASFV K205R protein

To develop a cELISA using Nb-HRP fusion protein as a probe, the Nb-HRP fusion protein was prepared according to the previously reported method with appropriately modifications [26]. The VHH target gene was amplified using pCANTAB 5E-VHH plasmid as a template, after digesting with *Eco*R I and *Nhe* I (NEB, Ipswich, MA, USA), the VHH gene was then inserted into the pCAGGS-HA-HRP vector. The recombinant plasmid was named pCAGGS-Nbs-HRP, which contained codon-optimized HRP gene sequence, His tag, multiple cloning sites and secretion signal sequence. The sequencing correct plasmids were transfected into HEK-293 T cells using polyetherimide reagents (PEI; Polysciences Inc., Warrington, PA. USA). At 48 h post transfection, cells and culture supernatants were collected for detection of Nbs-HRP fusion protein expression using indirect fluorescence assay (IFA) or direct ELISA. At the same time, the specificity and affinity of the Nbs-HRP fusion protein expressed by HEK-293 T cells to ASFV K205R protein were detected by direct ELISA.

For IFA, at 24 h post-transfection, the HEK-293 T cells were fixed with -20 °C pre-cold 70% alcohol and blocked with 1% BSA for 2 h at 37 °C. Subsequently, the cells were incubated overnight with mouse anti-HA monoclonal antibody as the first antibody at 4 °C and followed by washing three times with PBS. Then the cells were incubated with Alexa Fluor 594-conjugated goat anti-mouse IgG (H&L) for 1 h at 37 °C in the dark. After washing three times with PBS, nuclei were stained with DAPI for 5 min in the dark. Images were obtained using a fluorescence microscope (Leica AF6000; Leica Microsystems GmbH).

For direct ELISA, the ELISA plate was coated with K205R protein (400 ng/well) and incubated overnight at 4 °C. After blocking with 2.5% dry milk at room temperature (RT) for 1 h (200 μl/well), the plate was washed three times with PBS'T. 100 and 200 µL cell supernatants containing the Nbs-HRP fusion protein were incubated in ELISA plate for 1 h at 37 °C. Following washing three times with PBS'T, 100 μl/well TMB was added and incubated at 37 °C for 15 min in the dark. Next, 50 μl/well of 3 M H_2_SO_4_ was added to stop the reaction, and the absorbance of the each sample was detected at 450 nm using a spectrophotometer (BioTek Instruments, Winooski, VT, USA).

### Establishment of a competitive ELISA based on Nb-HRP probe

First, the optimal antigen coating concentration and dilution of Nb-HRP fusion protein were analyzed using a checkerboard titration assay by direct ELISA. Different concentrations of K205R protein (10, 20, 40, 80, 160, 320, and 640 ng/well) were coated into ELISA plates, and then the OD450 value was calculated when the Nb-HRP supernatants was diluted at ratios of 1:10, 1:100, 1:1000 and 1:10,000. When the OD450 absorbance value reached 1.0, the combination was determined as the optimal antigen coating concentration and the dilution ratio of Nb-HRP fusion protein. Three separate positive and negative pig serum were diluted at 1:5, 1:10, and 1:20 and detected using cELISA to determine the optimal dilution ratio of pig serum. According to the minimum ratio of OD450 value of positive serum and negative serum (P/N), the optimal serum dilution was determined. The optimal reaction time of the mixtures containing the Nb-HRP probe and the positive or negative serum with K205R protein were measured at 10, 30, and 50 min, respectively, followed with TMB imcubation for 10 and 15 min. Using a checkerboard titration method, the optimal incubation and colorimetric reaction time were determined when the minimum ratio of P/N value was obtained.

A novel cELISA was established after optimizing different reaction conditions: (1) ELISA plates were coated with the optimal concentration of K205R protein and incubated overnight at 4 °C. (2) After washing three times with PBS'T, the ELISA plates were blocked with 2.5% dried milk in PBS'T (200 µl/well) at RT for 1 h. (3) After washing three times with PBS'T, 100 µl of the optimal dilutions of serum sample and Nb-HRP supernatants mixtures in PBS'T was added to each well and incubated at RT for optimal times. (4) After washing three times with PBS'T, 100 µl/well of fresh TMB was added and incubated at 37 °C in the dark for the optimal duration. (5) 50 μl/well of 3 M H_2_SO_4_ was used as stop solution, and the absorbance value of each sample was detected at 450 nm.

### Cut-off value, sensitivity, specificity and repeatability analysis of the developed cELISA

The PI values were determined using the following formula: PI (%) = [1-(OD_450nm_ value of testing pig serum sample/ OD_450nm_ value of negative pig serum sample)] × 100%. The cut-off value was determined using 154 ASFV antibody-negative pig serum samples. To ensure a 99% confidence level of negative porcine serum samples within this range, the cut-off value was calculated using the mean PI value of the negative samples plus 3 standard deviation (SD) after testing the above 154 serum samples using the established cELISA.

To assess the sensitivity of the developed cELISA, 5 inactivated ASFV antibody-positive pig serum samples were doubling diluted (from 1:8 to 1:1024) and detected using the cELISA to determine the lowest detection dilution. At the same time, 89 inactivated ASFV-clinical positive sera were detected to analyze PI distribution as well.

The specificity of cELISA was assessed via analyzing PI distribution by testing 320 ASFV-clinical negative sera. Meanwhile, the cross-reacted assay was evaluated between the Nb-HRP and serum antibodies of some other common swine viruses, such as porcine reproductive and respiratory syndrome virus (PRRSV), porcine epidemic diarrhea virus (PEDV), porcine pseudorabies virus (PRV), classical swine fever virus (CSFV) and porcine circovirus type 2 (PCV2).To evaluate the repeatability of the developed cELISA, 8 separate ASFV antibody-positive and -negative pig serum were detected and the inter-polate (between plates) and intra-plate (within a plate) variabilities were analyzed. The coefficient of variation (CV) was used to evaluate inter-plate variation and intra-plate variation. To calculate the inter-plate CV, each serum sample was detected using three different ELISA plates, while for the intra-plate CV, three replicates within each ELISA plate were detected.

### Evaluation of agreement between the developed cELISA and commercial ELISA kits

Two hundred eighty-five clinical pig serum samples were detected using the developed cELISA and commercial ELISA kit, separately, and analyzed using SPSS software to evaluate the clinical agreement of the two detection method.

### Statistical analysis

Statistical analysis was performed using GraphPad Prism version 5.0 (GraphPad Software, San Diego, CA, USA). Data are expressed as the mean ± SD. Repeatability was assessed using CV (CV = SD/mean), where a CV value less than 15% for the intra-plate assay was considered an acceptable repeatability level for the assay. Kappa values were calculated using SPSS software version 20 (IBM Corp.; http://www.spss.com.cn).

## Results

### Expression and purification of recombinant ASFV K205R Protein

**Fig. 1:**
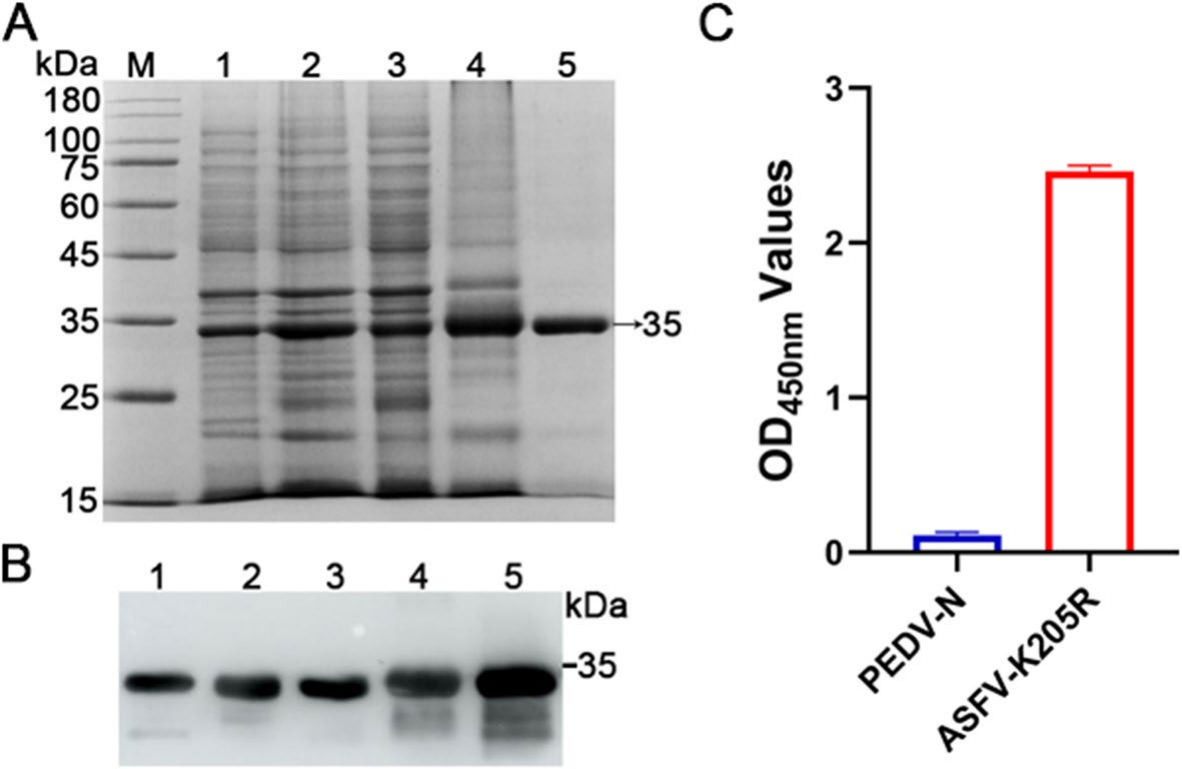
Expression and purification of K205R-His protein. **A** The K205R-His protein was analyzed by SDS-PAGE. **B** and **C** Western blotting and iELISA analysis of the antigenic property of the K205R protein using inactivated ASFV antibody-positive serum as the primary antibody. Lane 1: pET30a empty vector control; Lane 2: IPTG, 0.5 mM; Lane 3: Supernatant soluble K205R protein; Lane 4: Inclusion body of K205R protein; Lane 5: Purified K205R protein

The recombinant His-tag K205R protein was successfully expressed in inclusion bodies of the expected size of 35 kDa. Then, the K205R protein was affinity purified using a Ni–NTA resin, and SDS-PAGE showed that the K205R protein was obtained with high purity (Fig. 1A). Next, the purified K205R protein was used to determine the antigen reactivity by western blotting and iELISA. As shown in Fig. 1B and C, the K205R protein could react with the inactivated ASFV-positive pig sera. After buffer exchange to PBS, the concentration of the purified K205R protein was determined and would be used as the antigen to screen specific nanobodies and establish cELISA.

### Construction of VHH library

**Fig. 2:**
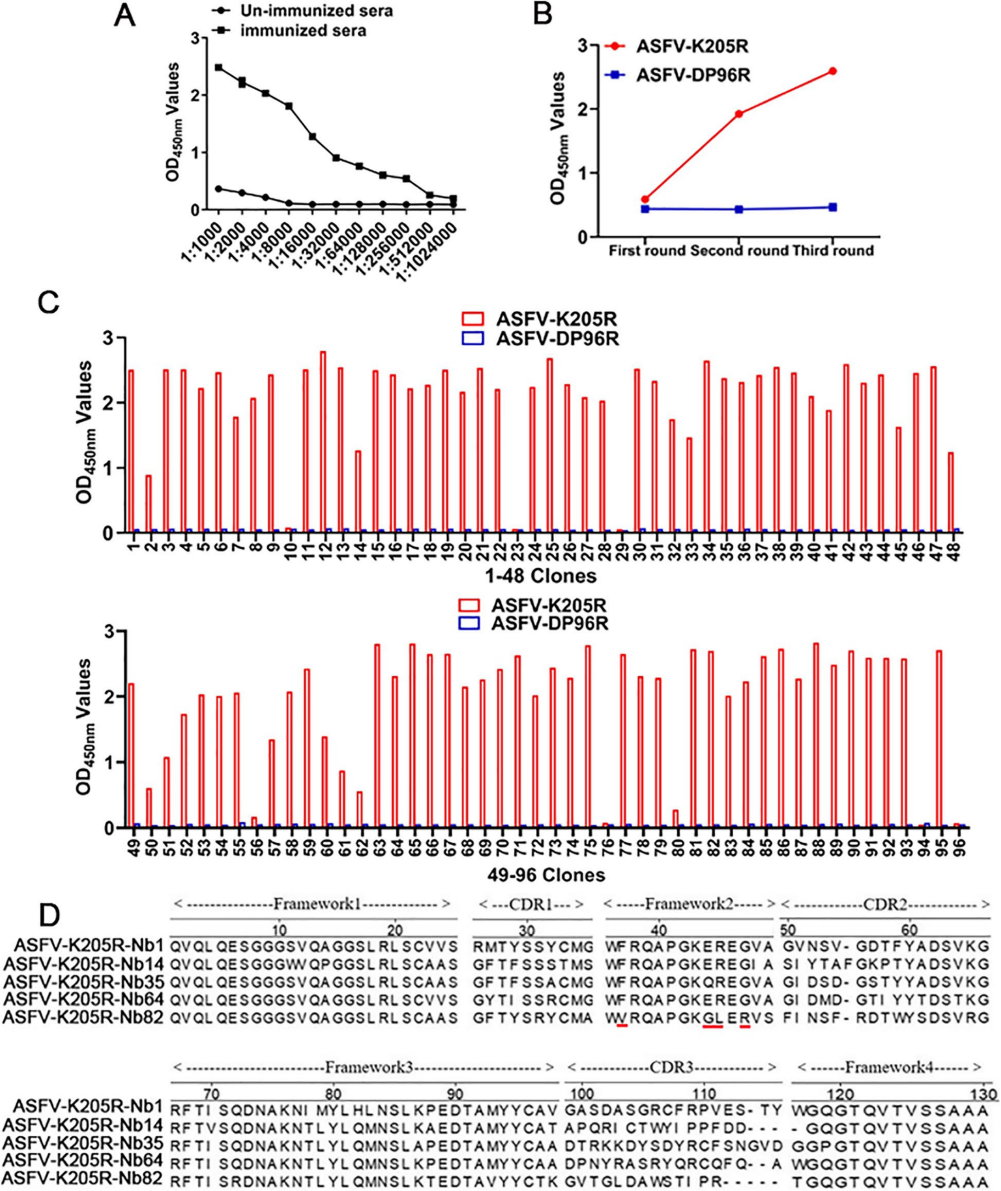
Screening of K205R specific nanobody. **A** iELISA analysis of serum antibody titer against K205R protein of the immunized camel. **B** ELISA analysis the enrichment of specific phage. **C** iELISA analysis of periplasmic extracts from 96 clones that specifically bind to K205R protein. **D** Five K205R specific nanobodies were screened according to amino acid sequence alignment. The red line indicates the hallmark residues at 37, 44, 45, and 47

After the last immunization, the serum titer against K205R protein reached 1:256,000 according the iELISA (Fig. 2A), suggesting that the camel immunization was succeed. Then, following RNA extraction, cDNA amplification, ligation and transformation, a phage-display VHH library consisting of approximately 5 × 10^8^ colonies was successfully constructed. Colony PCR analysis of positive rate reflected that 92% of these colonies contained the expected VHH genes. 50 clones were then randomly selected for sequencing analysis. The results suggested that each clone had a typical VHH sequence (data not shown), indicating that the diversity and quality of the library was good.

### Screening of specific nanobodies against ASFV K205R protein

Three rounds of bio-panning were performed to select nanobodies that specifically bind to K205R protein. After three rounds of bio-panning, VHHs phage particles specific for K205R protein were efficiently enriched (Fig. 2B), and the ratio of positive clones to negative clones (P/N) increased from 2.3 to 114 (Table 1). Then, 96 individual clones were picked from the products of the third round bio-panning plate randomly, and the periplasmic extracts were extracted to screen the specific extracts binding with K205R protein. Out of the 96 colonies, 88 were specifically bound with the K205R protein (Fig. 2C). Next, the 88 colonies were sequenced, and based on the CDR3 hypervariable region sequence, 5 different K205R-specific nanobodies were finally screened (K205R-Nb1, -Nb14, -Nb35, -Nb64, -Nb82) (Fig. 2D).

**Table 1:** Enrichment of nanobodies against ASFV K205R protein specific phages during three rounds of panning

Round of panning	Input (PFU/Well)	P output (PFU/Well)	N output (PFU/Well)	Recovery rate	Enrichment(P/N)
First round	5 × 10^10^	1.26 × 10^5^	5.4 × 10^4^	2.52 × 10^–6^	2.3
Second round	5 × 10^10^	6.5 × 10^6^	7.4 × 10^4^	1.3 × 10^–4^	88
Third round	5 × 10^10^	7.4 × 10^7^	6.5 × 10^5^	1.48 × 10^–3^	1.14 × 10^2^

### Identification of specific nanobodies against ASFV K205R Protein

**Fig. 3:**
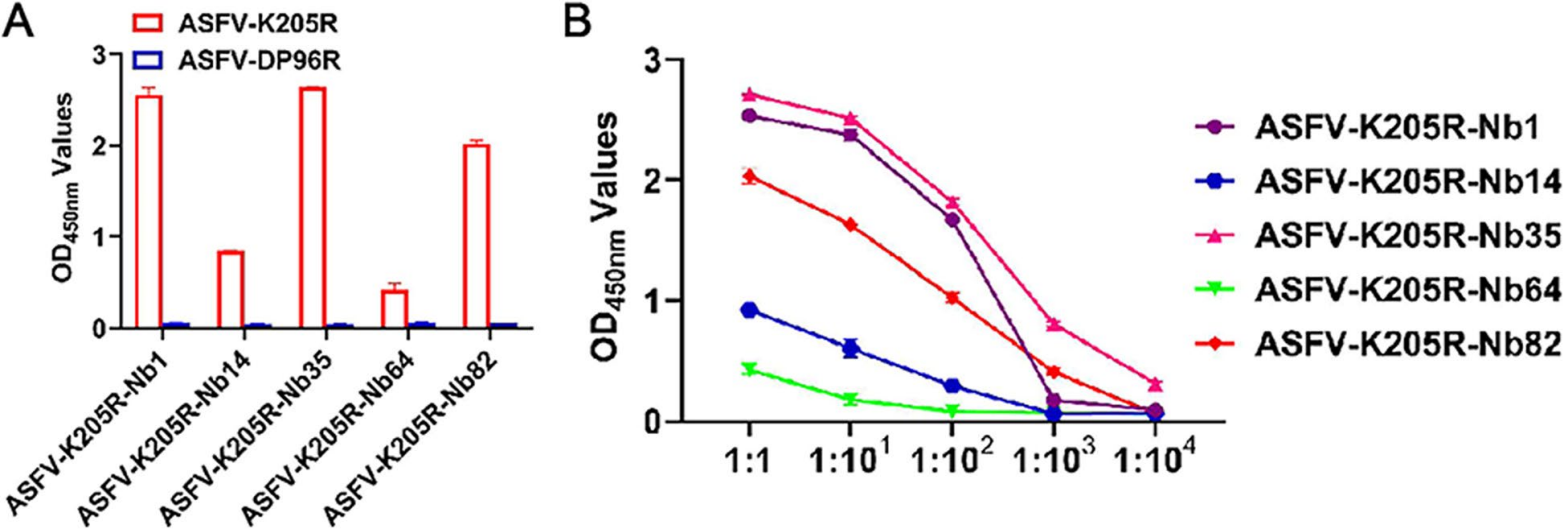
Identification of the nanobodies against the K205R protein. **A** iELISA identification of nanobodies specifically recognized K205R protein. ASFV DP96R protein was used as a negative control with the same expression system as K205R. **B** Affinity analysis of nanobodies in cytoplasmic extracts for K205R protein

To analyze the specificity and affinity of nanobodies against K205R protein, iELISA was implemented. iELISA results showed that the five nanobodies reacted with recombinant K205R protein but did not react with ASFV DP96R protein (Fig. 3A). The His-tagged DP96R protein was also expressed and purified using the pET-30a vector and Ni–NTA column, which effectively ruled out the unspecific binding activiry of the nanobodies, such as those recognizing the His-tag. Titer analysis of K205R-Nb1, -Nb35, and -Nb82 periplasmic extracts showed that they exhibited higher titers than that of K205R-Nb14 and -Nb64 (Fig. 3B).

### Production of K205R-Nbs-HRP fusion protein

**Fig. 4:**
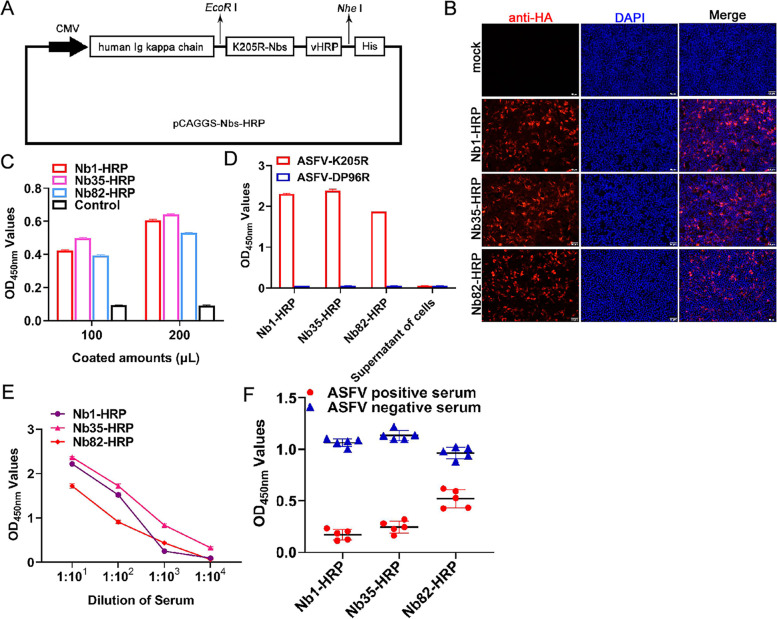
Secretory expression and functional analysis of Nbs-HRP in HEK-293 T cell culture supernatants. **A** Schematic representation of Nbs-HRP fusion protein construction. **B** IFA identification of Nbs-HRP expression in HEK-293 T cells using anti-HA monoclonal antibody. **C** Detection of secretory expression of Nbs-HRP fusion protein in HEK-293 T cell culture supernatants. **D** Direct ELISA analysis of Nbs-HRP specifically reacted with K205R protein. ASFV DP96R was used as a control. **E** Titration of the Nbs-HRP binding with the K205R protein. **F** cELISA comparative analysis of Nb1, Nb35 and Nb82 blocking capability of the binding of ASFV antibody-positive serum to K205R protein

For expression of K205R-Nbs-HRP fusion proteins, HEK-293 T cells were transfected with pCAGGS-Nb1-HRP, -Nb35-HRP, and -Nb82-HRP plasmids respectively, and cell samples were subjected to IFA and direct ELISA analysis (Fig. 4A). As shown by the IFA and direct ELISA results, K205R-Nb1-HRP, -Nb35-HRP and -Nb82-HRP were all expressed and secreted into the cell culture supernatant successfully (Fig. 4B and C). All of the three Nbs could specifically bind to the K205R protein, and displayed high affinity, according to the direct ELISA results (Fig. 4D and E). Based on the specificity and affinity analysis results, Nb1, Nb35, and Nb82 were selected for cELISA analysis further. The cELISA results showed that all three nanobodies could block the reaction of inactivated ASFV-positive serum with K205R protein, however, Nb1 had the highest blocking rate (Fig. 4F). Therefore, Nb1-HRP fusion protein was prepared with Nb1 and used as a sensitive probe to develop a cELISA detection method for ASFV serum antibodies.

### Development of cELISA Using Nb1-HRP Fusion protein as a reagent

**Fig. 5:**
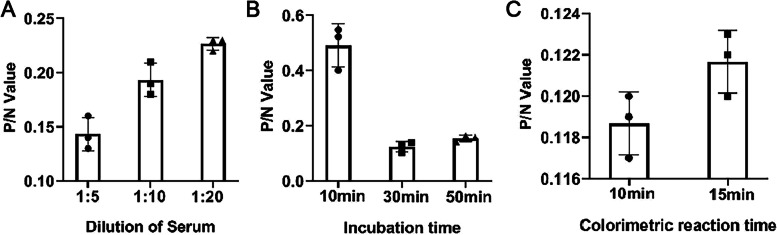
Determination of optimal reaction conditions of the cELISA. **A** cELISA analysis of the optimal dilution ratio of pig serum. **B** Determination of optimal competition time for K205R protein binding between ASFV antibody-positive serum and Nb1-HRP fusion protein. **C** Optimal colorimetric reaction time of the developed cELISA

Checkerboard titration was used to determine the optimal coating concentration of K205R protein and the dilution of Nb1-HRP fusion protein. The results showed that the optimal K205R protein coating concentration was 320 ng/well, and the best Nb1-HRP dilution was 1:100 (Table S2). According to different dilutions of the 3 positive and negative serum yielding the lowest P/N, the optimal dilution of pig serum sample to be tested was 1:5 (Fig. 5A). The optimal incubation time of pig serum to be tested and Nb1-HRP supernatants was 30 min, and the optimal colorimetric reaction time was 10 min, since the P/N value at this time was the smallest (Fig. 5B and C).

### Cut-off values for the cELISA

One hundred forty-seven standard ASFV antibody-negative serum samples were detected using the developed cELISA to determine the cut-off value. The results showed that the average PI ($\overline{x }$) of the negative serum samples was 5.44%, and the SD was 9.8%. Thus, the cut-off value for the developed cELISA was 34.8%. The sample was regarded as ASFV antibody-positive at a PI ≥ 34.8% and ASFV antibody-negative at a PI < 34.8%.

### Determination of the specificity, sensitivity and reproducibility of the cELISA

**Fig. 6:**
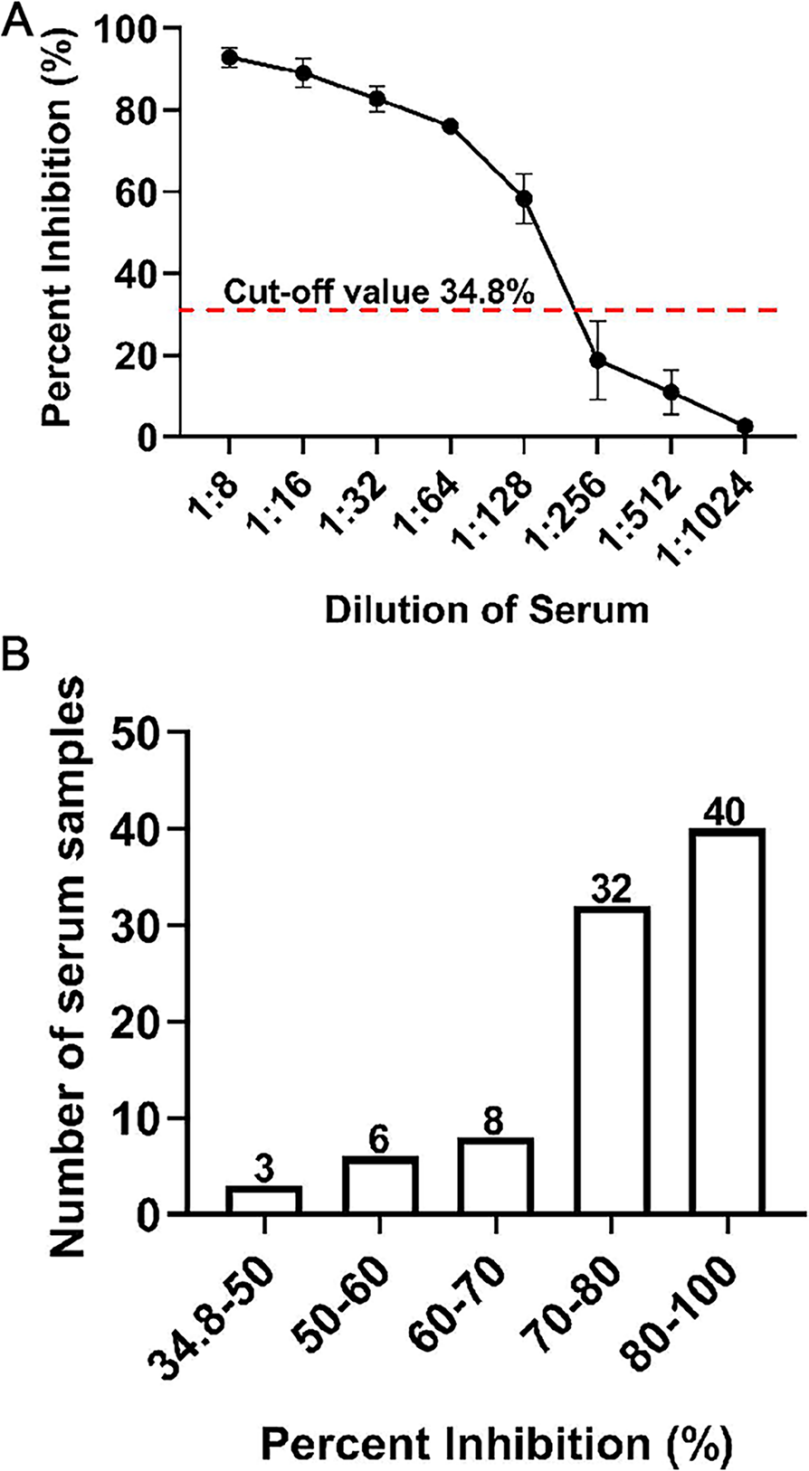
Sensitivity analysis of the developed cELISA. **A** Determination of the maximum dilution of inactivated ASFV antibody-positive pig serum. **B** Distribution of the PI values by testing the positive clinical sera for anti-ASFV antibodies using cELISA

Different dilutions of inactivated ASFV antibody-positive pig serums were used for cELISA detection to determine its sensitivity. The results of cELISA showed that serum at a dilution of 1:128 were ASFV antibody-positive, and serum at a dilution of 1:256 were ASFV antibody-negative (Fig. 6A), suggesting that the developed cELISA had a high sensitivity. In addition, 89 samples of ASFV antibody-clinically positive pig serum were all positive by the cELISA, of which 72 samples had PI values greater than 70%, whereas only 3 samples had PI values between 34.8% and 50% (Fig. 6B). Therefore, the sensitivity of the developed cELISA in the present study to detect clinical pig serum was 100%.

**Fig. 7:**
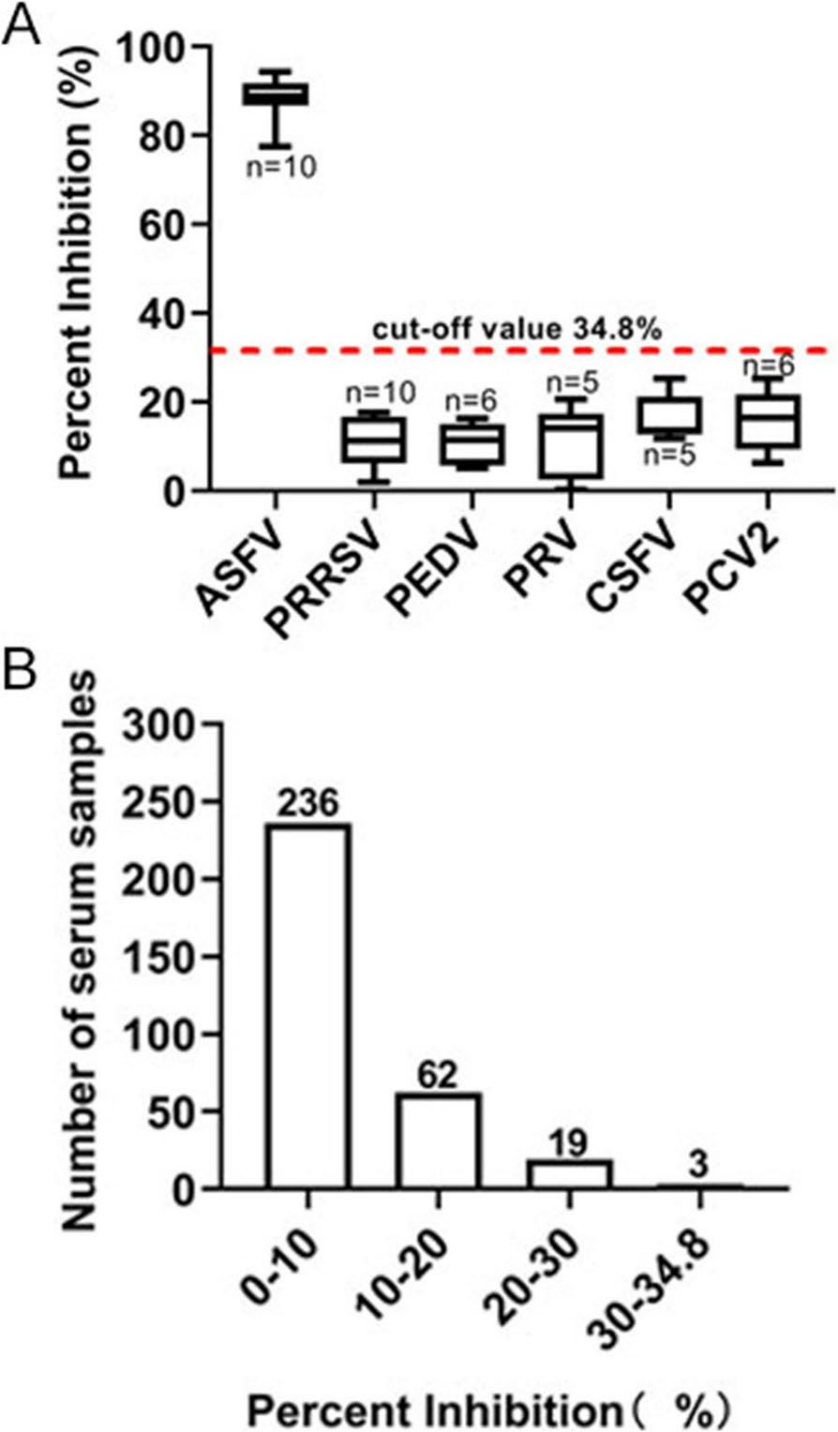
Specificity analysis of the developed cELISA. **A** Specificity analysis of the developed cELISA by detection of PRRSV-, PEDV-, PRV-, CSFV-, and PCV2-antibody positive serum. **B** ASFV antibody-negative serum samples were tested using the cELISA to analyze distribution of the PI values

To determine the specificity of the cELISA, positive serum collected from other common swine viral diseases, including PRRSV, PEDV, PRV, CSFV and PCV2, were detected to determine the cross-reactivity. As shown in Fig. 7A, inactivated ASFV antibody-positive serum displayed PI values of 75%-91%, while other swine virus-positive serum displayed PI values of 1%-20%, indicating that the specificity of the cELISA was excellent. In addition, 320 ASFV-antibody negative pig serum samples were all tested negative using the developed cELISA, of which 298 serum samples had PI values less than 20%, whereas only 3 samples had PI values between 30% and 34.8% (Fig. 7B).

To analyze the repeatability of the cELISA, intra- and inter-plate variability were evaluated using 8 clinical pig serum samples. The intra-plate CV range of PI was 2.23%-8.45%, the median value was 5.34%, and the inter-plate CV range of PI was 3.71%-10.90%, the median value was 7.31% (Table 2), suggesting a good repeatability of the developed cELISA method.

**Table 2:** Reproducibility of the cELISA determined by CV % value of intra and inter assay

	**CV % value range of 8 serum samples**	**Median value**
Intra assay precision (CV%)	2.23–8.45	5.34
Inter assay precision (CV%)	3.71–10.90	7.31

*Note*: Intra assay precision: Determined from three repetitions (well-to-well) of 8 serum samples in the same method. Inter assay precision: Determined from three repetitions (plate-to-plate) at different time

### Clinical application

**Fig. 8:**
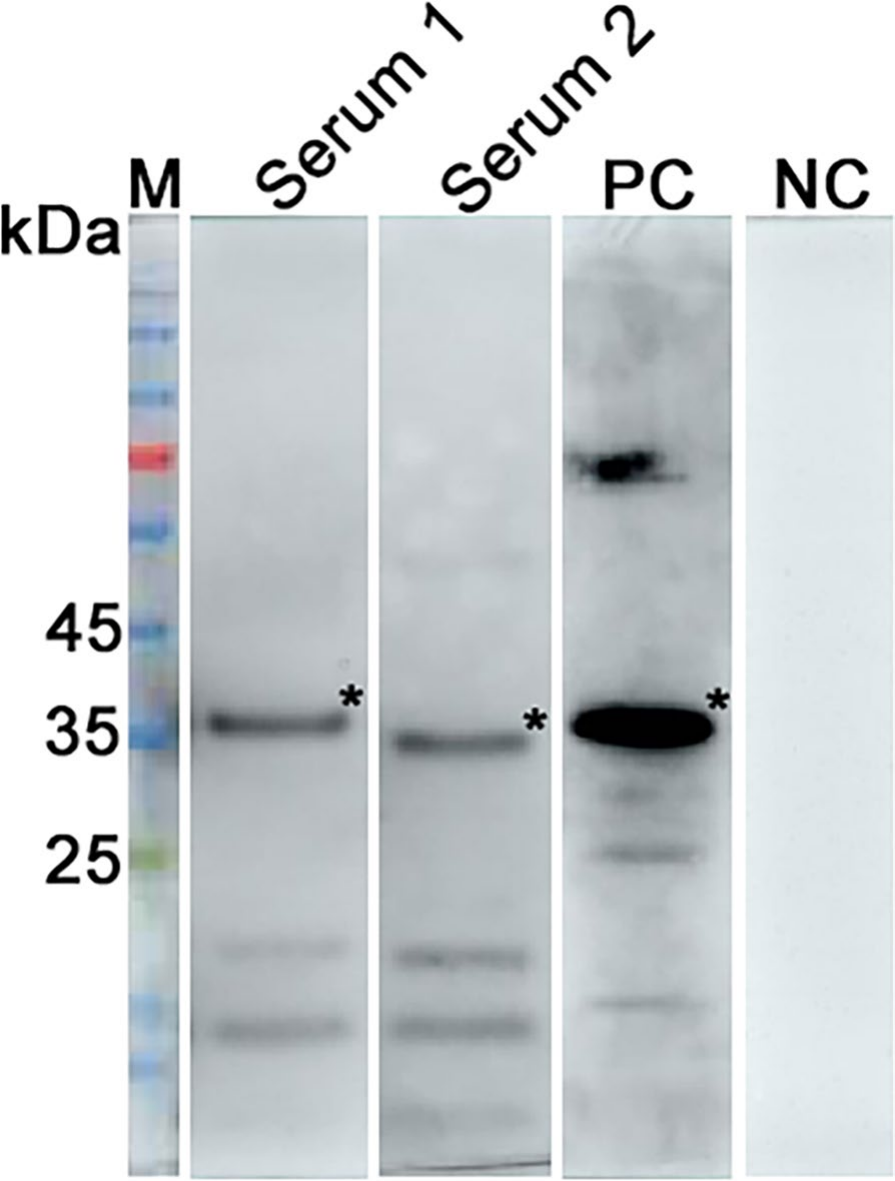
Evaluation of the discrepancy of test results between the cELISA and commercial ELISA kit. Western blotting of the 2 swine serum samples with a discrepant result between the developed cELISA and commercial ELISA kit. A negative serum and a inactivated standard ASFV antibody-positive serum were used simultaneously as the control. PC: positive serum control; NC: negative serum control

Two hundred eighty-five inactivated clinical pig serum collected from pig farms in Henan and Hebei provinces were analyzed using the developed cELISA and the commercial ELISA kit to assess the former clinical application value. The developed cELISA detection results revealed that among these serum samples, 24 were ASFV antibody-positive serum samples and 261 were ASFV antibody-negative serum samples, while the commercial ELISA kit showed that 22 were ASFV antibody-positive serum samples and 263 were ASFV antibody-negative serum samples. In general, among the 285 serum samples detected, 283 serum samples showed consistent results by the two methods (22 + /261-), the agreement rate was 99.3% (Kappa = 0.98). According to the statistical analysis results, the developed cELISA exhibited high agreement with the commercial ELISA kit, and there was no significant difference between the two kits (Kappa > 0.4) (Table 3). Next, the 2 serum samples with discordant test results were further analyzed by western blotting. All 2 were found to be positive, since the K205R band was clearly detected in the 2 samples, which was in accordance with the developed cELISA (Fig. 8), indicating higher sensitivity of the developed cELISA. Similar band were obtained when using inactivated standard ASFV antibody-positive serum as a positive control (Fig. 8). In conclusion, the developed cELISA has good sensitivity and specificity, and has high agreement with commercial ELISA kits, which can be used for clinical detection.

**Table 3:** Comparisons of the developed cELISA with commercial ELISA kit by detecting clinical pig serum samples

Samples	cELISA	Number	Commercial ELISA Kit		Agreement (%)	Kappa value
			** + **	**-**		
**Clinical sera**	+	24 (A)	22 (B)	2	99.3%	0.98
	-	261 (C)	0	261 (D)		

Agreement (%) = (B + D) / (A + C)

The kappa value > 0.4 was regarded as significant difference

## Discussion

ASFV, one of China's most devastating emerging swine pathogens, causes nearly 100% mortality in naive herds [29]. Currently, no effective treatments or vaccines are available to combat ASFV, and disease control relies solely on early detection and implementation of strict sanitary procedures [30]. Therefore, it is particularly important to establish a fast and efficient detection method for ASFV. ELISA is suitable for large-scale clinical epidemiological investigations [31]. Antibodies are considered one of the most effective reagents for developing sensitive and specific ELISA [32, 33]. Based on the advantage of easy genetic manipulation of nanobody, K205R protein-specific Nb-HRP fusion protein was used as a probe to develop immunoassays for ASFV antibody detection in this research.

Currently, there is no de facto gold standard for ASFV detection. Commercial ELISA kits have been developed, but the need for high-purity antigens and enzyme-labeled secondary antibodies greatly increases production costs and limits clinical applications. Compared with traditional ELISA, the developed cELISA based on Nb-HRP fusion protein in this study exhibits some unique advantages, such as simple production process and low-costing. Secondly, compared with traditional antibodies, the ratio of nanobodies to HRP is 1:1 when nanobodies are fused and expressed with HRP, while traditional antibodies can not achieve a 1:1 effect. The cELISA established with Nb-HRP as the probe is more sensitive than HRP-labeled traditional antibodies. The developed cELISA does not require the addition of an enzyme-labeled secondary antibody, which saves time and effort, mainly saves costs, and can be widely used in clinical testing. Due to laboratory biosafety restrictions, experimental swine challenge were not performed in this study, thus it is not feasible to determine the sensitivity of the developed cELISA under experimental challenge conditions, and the sensitivity difference between the developed cELISA and the commercial ELISA kit requires further study in the future for a more comprehensive assessment.

The host immune response to ASFV infection is sophisticated, including cellular and serological responses [34]. A systematic analysis found that ASFV has 12 proteins with principal serological epitopes, among which the 4 proteins p54, A104R, B602L, and K205R produce higher antibody titers and last longer, indicating that they have great potential as effective diagnostic tools for ASF [35]. The amino acid sequence of K205R is conserved, and in the early stage of virus infection, the K205R gene is the first to be replicated in large quantities, and the antibody is produced early [24]. These provide a strong guarantee for establishing rapid and timely detection of ASFV. In addition, some studies have also reported that ASFV proteins such as p54, p30 and p72 were used for ASFV serum antibody detection [13, 36, 37]. The difference of sensitivity and specificity between using K205R protein as antigen and other antigens are still unclear. However, considering that some ASFV proteins, such as p72, appear in the middle and late stages of viral infection [38], these traditional tests may be more inclined to determine whether pigs are ASFV-antibody positive or negative, and the cELISA developed in this study may be more sensitive than traditional methods, however, this conjecture needs further verification. Although the K205R protein can induce antibodies in pigs in the early stage of ASFV infection, whether these antibodies have virus-neutralizing activity or protective effect in vivo is still unclear. Considering that K205R can synthesize and induce antibodies in the early stage of virus infection, it can still be regarded as an effective diagnostic indicator for early ASFV infection. Anyhow, the high-sensitivity early detection of ASFV-infected or suspected infected pigs may provide an effective reference for preventive immunization or other biosafety measures to prevent further spread of infection.

## Conclusion

In conclusion, a novel ELISA for ASF serum antibody detection with simple operation and low cost was developed using Nb-HRP fusion protein as an effective tool in this study. This method has the potential to be developed into a commercial ELISA kit, which may provide new options for the prevention and control of ASFV.

## Supplementary Information

**Additional file 1:** **Table S1.** Primers used in this study. **Table S2.** Optimized amount of ASFV K205R protein as the coating antigen anddilution of Nb1-HRP fusions using the direct ELISA.

## Data Availability

All data and materials presented in the study are available from the corresponding author on reasonable request.

## Abbreviations

ASFV: African swine fever virus
ELISA: Enzyme linked immunosorbent assay
Nb: Nanobody
Nb-HRP: Nanobody-horseradish peroxidase
cELISA: Competitive ELISA
PCR: Polymerase chain reaction
RT-qPCR: Reverse transcript-quantitative polymerase chain reaction
IPTG: Isopropyl β-D-1-thiogalactopyranoside
iELISA: Indirect ELISA
VHH: Variable domain of the heavy-chain of heavy-chain antibody
IFA: Indirect fluorescence assay
RT: Room temperature
PI: Percent inhibition
SD: Standard deviation
CV: Coefficient of variation
