# Supplementary material for: K205R specific nanobody-horseradish peroxidase fusions as reagents of competitive ELISA to detect African swine fever virus serum antibodies
Source: BMC Vet Res. 2022 Aug 20;18:321. doi: 10.1186/s12917-022-03423-0 (PMC9392344; doi:10.1186/s12917-022-03423-0)

**Table S1 Primers used in this study**

| **Primers** | **Sequence (5’- 3’)** | **Usage** |
| --- | --- | --- |
| ASFV-K205R-F  ASFV-K205R-R | CGC*GGATCC*ATGGTTGAGCCACG  CCG*CTCGAG*TTACTTCTTCATC | pET30a-ASFV-K205R  Overlap-VHH |
| VHH-F1 | GTCCTGGCTGCTCTTCTACAAGG |  |
| VHH-R1 | GGTACGTGCTGTTGAACTGTTCC |  |
| VHH-F2 | CAGGTGCAG*CTGCAG*GAGTCTGGGGGAGR |  |
| VHH-R2 | CTAGT*GCGGCCGC*TGAGGAGACGGTGACCTGGGT |  |
| Nb-F | CCG*GAATTC*ATGGAGACCGACACC | pCAGGS-Nbs-HRP |
| Nb-R | CTA*GCTAGC*TTAGTGGTGATGGTG |  |

Note: Restriction sites are underlined

**Table S2 Optimized amount of ASFV K205R protein as the coating antigen and dilution of Nb1-HRP fusions using the direct ELISA**

| **Nb1-HRP** | **OD_450_ values after different antigen coating concentration (ng/100μL)** | | | | | | |
| --- | --- | --- | --- | --- | --- | --- | --- |
|  | **10** | **20** | **40** | **80** | **160** | **320** | **640** |
| **1:10** | 0.0798 | 0.121 | 0.1629 | 0.3281 | 1.319 | 2.2079 | 2.5156 |
| **1:100** | 0.0581 | 0.0986 | 0.1553 | 0.2457 | 0.8533 | 1.073 | 1.408 |
| **1:1000** | 0.0321 | 0.0852 | 0.121 | 0.2153 | 0.2957 | 0.8053 | 0.898 |
| **1:10000** | 0.036 | 0.049 | 0.034 | 0.049 | 0.075 | 0.056 | 0.063 |

Note: The optimal amount of ASFV K205R protein and dilution of Nb1-HRP fusions were selected when the OD_450nm_ values of the direct ELISA was approximately 1.0.

Original uncropped western blot image of Figure 1B.


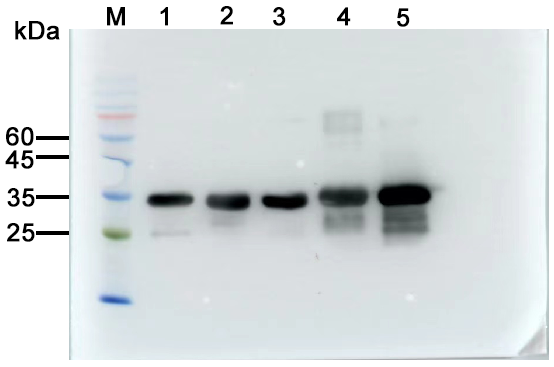


M: protein marker; Lane 1: pET30a empty vector control; Lane 2: IPTG, 0.5 mM; Lane 3: Supernatant soluble K205R protein; Lane 4: Inclusion body of K205R protein; Lane 5: Purified K205R protein.

Original uncropped western blot image of Figure 8.


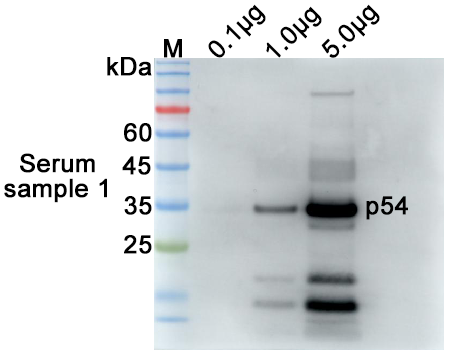

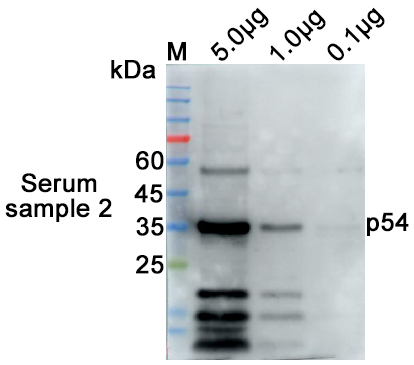


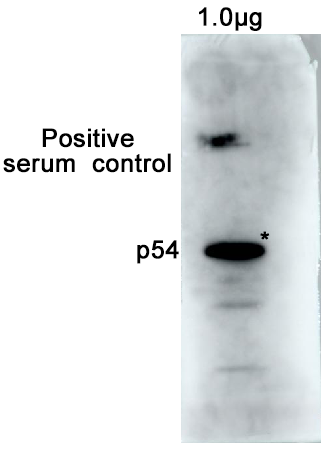

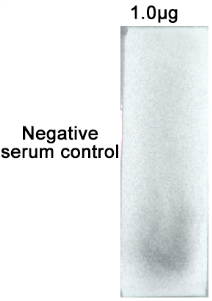

Supplement: Supplementary file 1 — Additional file 1: Table S1. Primers used in this study. Table S2. Optimized amount of ASFV K205R protein as the coating antigen anddilution of Nb1-HRP fusions using the direct ELISA. [file 12917_2022_3423_MOESM1_ESM.docx]
